# Ergonomics in the operating room and surgical training: a survey on the Italian scenario

**DOI:** 10.3389/fpubh.2024.1417250

**Published:** 2024-08-07

**Authors:** Stefano Restaino, Marco D’Indinosante, Federica Perelli, Martina Arcieri, Vittorio Cherchi, Marco Petrillo, Anna Franca Cavaliere, Stefano Cianci, Giulia Pellecchia, Roberto Luca Meniconi, Alessandro Coppola, Vito Chiantera, Giovanni Scambia, Lorenza Driul, Giuseppe Vizzielli, SPIGC Working Group, Federico Berton

**Affiliations:** ^1^Clinic of Obstetrics and Gynecology, “S. Maria della Misericordia” University Hospital, Azienda Sanitaria Universitaria Friuli Centrale (ASUFC), Udine, Italy; ^2^Dipartimento per le Scienze Della Salute Della Donna, del Bambino e di Sanità Pubblica, UOC Ginecologia Oncologica, Fondazione Policlinico Universitario Agostino Gemelli IRCCS, Rome, Italy; ^3^Division of Gynaecology and Obstetrics, Santa Maria Annunziata Hospital, USL Toscana Centro, Florence, Italy; ^4^General Surgery Clinic and Liver Transplant Center, University Hospital of Udine, Udine, Italy; ^5^Gynecologic and Obstetric Clinic, Department of Medicine, Surgery and Pharmacy, University of Sassari, Sassari, Italy; ^6^Department of Gynecology and Obstetrics of "San Giovanni Calibita" Fatebenefratelli Hospital-Gemelli Hospital, Rome, Italy; ^7^Dipartimento di Ginecologia Oncologica e Chirurgia Ginecologica Miniinvasiva, Università degli studi di Messina, Policlinico G. Martino, Messina, Italy; ^8^Department of Medicine, University of Udine, Udine, Italy; ^9^Department of General Surgery and Liver Transplantation, San Camillo Forlanini Hospital, Rome, Italy; ^10^Department of General Surgery, Sapienza Università di Roma, Rome, Italy; ^11^Unit of Gynecologic Oncology, National Cancer Institute, IRCCS, Fondazione "G. Pascale", Naples, Italy

**Keywords:** surgery, ergonomic, gyne and obstetrics, operatory room, survey

## Abstract

**Introduction:**

Surgical-related injuries are frequent, in fact the reported percentage of musculoskeletal disorders in surgeons is between 47% and 87%. These conditions are caused by long periods of standing, incorrect postures, repeated movements, little rest between operations, the lack of integrated operator rooms, the correct number and arrangement of monitors and the use of non-ergonomic instruments. This survey aims to assess the Italian overview both highlighting how prevalent surgical-related injury is in our surgeons and whether there is an operating room ergonomics education program in Italian surgical specialty schools.

**Methods:**

An anonymous questionnaire was designed through SurveyMonkey© web application. This survey was composed of 3 different sections concerning the general characteristics of the participants, their surgical background and any training performed, and any injuries or ailments related to the surgical activity. The survey was carried out in the period 1th of December 2022 and the 6th of February 2023.

**Results:**

At the close of our survey, 300 responses were collected. Among the participants, the two most represented specialties were Gynecology and Obstetrics (42.3%) and General Surgery (39.7%) and surgeons were mainly employed in the Northern regions of Italy (54.8%). Analyzing the participants’ background, 61.7% of the respondents had laparoscopic training during their training and only 53.1% had a pelvic trainer during their residency. In accordance with 98.7% of the respondents, during surgery we have the feeling of being in an uncomfortable position that causes discomfort or muscle pain, and regarding the frequency of these discomforts, the majority of our study population experiences these problems monthly (46.2%), while in 29.6% it is experienced weekly, 12.1% annually and finally 12.1% daily. The surgical approach that is most correlated with these disorders is laparoscopy (62.7%) while the one that causes the least discomfort is robotic surgery (1.4%). These discomforts cause 43.9% of our population to take a break or do short exercises to reduce pain during surgery, and the body areas most affected are the back (61.6%), neck (40.6%) and shoulders (37.8%).

**Conclusion:**

Despite this, our survey allows us to highlight some now-known gaps present in the surgical training program of our schools and the lack of protection toward our surgeons during their long career.

## 1 Introduction

Over the past years, minimally invasive surgery (laparoscopic or robotic) has gained wide acceptance in gynecology compared to the laparotomic approach due to its surgical as well as clinical benefits for patients, such as reduced operating time, less intra-operative blood loss, shorter postoperative hospital stay, lower rates of postoperative fever and infection and finally a faster patient recovery (1–3).

While such surgery is certainly a benefit for our patients, the emergence and spread of minimally invasive surgery has introduced ergonomic risks for surgeons (for example, including instrument length and handle design, improper monitor position, and excessively high operating tables) (4) and, although this issue is well known, the safety and health of physicians have received less attention than that of patients (5).

Surgical-related injuries are frequent, in fact the reported percentage of musculoskeletal disorders in surgeons ranges between 47% and 87% (5–11). These conditions are caused by long periods of standing, incorrect postures, repeated movements, little rest between operations, the lack of integrated operator rooms, the incorrect number and arrangement of monitors and the use of non-ergonomic instruments (12–15). These situations are also exacerbated by the *altruistic* attitudes of surgeons who often maintain incorrect postures for long periods to benefit their patients’ health or to increase their productivity, to the detriment of their own (15–17).

To reduce these conditions, there has been a growing interest in implementing strategies that reduce work-related injuries in surgeons (15).

Implementing ergonomic guidelines involves several strategies aimed at minimizing musculoskeletal disorders risks:

- Adjustable Equipment: operating room tables and surgical instruments should be adjustable to accommodate different body sizes and postures. Tables should be set at a height that allows the surgeon to maintain a neutral spine position without bending or stretching excessively (18).
- Neutral Body Posture: Surgeons and OR staff should maintain a neutral body posture, which includes keeping the neck in a neutral position, the back straight, and avoiding rotation or tilt of the torso. Shoulders should be relaxed, and elbows kept close to the body (19).
- Regular Breaks and Microbreaks: Implementing scheduled breaks and microbreaks can help reduce physical strain. During these breaks, stretching exercises and movements to relieve tension in specific muscle groups are recommended (20).
- Ergonomic Training Programs: Regular training on ergonomic principles should be provided to OR staff. This training can include proper lifting techniques, posture correction, and the use of ergonomic tools and equipment (20).

Despite our knowledge of the problem and our efforts, to date an educational program in surgical schools regarding proper ergonomics in the operating room is still not widely available. In fact, not all surgeons are aware of the existence of guidelines on ergonomics in the operating room and ergonomics education programs are not yet firmly established within surgical schools (21, 22).

From this background, we wanted to assess the Italian overview both by highlighting the prevalence of surgical-related injury in our surgeons and by investigating the existence of an operating room ergonomics education program in Italian surgical residency programs.

## 2 Materials and methods

We designed an electronic anonymous questionnaire on SurveyMonkey© web application (SVMK Inc., One Curiosity Way, San Mateo, United States) (23). The aim of this survey was explained to all participants with a brief introduction. Participation to the survey was voluntary, and no incentives were offered. No institutional review board approval was required.

The survey was composed of three different sections (Supplementary material S1). The first section included questions regarding biographical information, one’s role (physician in specialty training, structured physician, or other), one’s place of employment, and one’s surgical specialty. The second section focused on type and frequency of surgical activity routinely performed, on laparoscopic surgical training, whether surgical training would have been useful in their surgical formation, and what tools are used to increase their skills.

The last section dealt with ergonomics in the operating room, the occurrence and frequency of surgical practice-related injuries, the correlation between ailments and the organization/arrangement of one’s operating room, and whether these injuries affected personal life or required specific medical treatments for their resolution or improvement.

The study was conducted from 1th of December 2022 to the 6th of February 2023 and promoted with a mailing list, instant message services, and through the main social media Official account of the authors on Facebook, Instagram, and Linked-in, thanks to the support of the Italian Polyspecialistic Society of Young Surgeons (SPIGC).

Italian surgeons coming from any surgical specialty and attending any year of the training program were considered eligible for the survey’s analysis. The eligibility has no relation to the residents’ curricular activities. All participants were informed that the results of the survey would have been used for further statistical evaluation and scientific publication. Anonymity was guaranteed by study design.

After the closing date for questionnaire submissions, results were downloaded as a CSV (comma-separated values) file to be analyzed via Excel (Microsoft Corporation, Redmond, United States).

Results of the survey were reported according to the CHERRIES Guidelines (24).

## 3 Results

At the close of our survey, 300 responses were collected. Among the participants, the two most represented specialties were Gynecology and Obstetrics (42.3%) and General Surgery (39.7%) (Table 1) and surgeons were mainly employed in the Northern regions of Italy (54.8%; Table 2). The characteristics of the participants are summarized in Table 3. 50.8% were men while 49.2% were women; the most represented age group was between 31 and 40 years (46.2%) while 46% of the population were surgeons in training while 54% were already specialized physicians. 41.8% had performed less than 50 operations in the last 12 months, while 28.1% had performed between 50 and 100 operations and 30.1% more than 100 operations. Lastly, the number of surgeons with more than 150 procedures as first operator was 95 (36.0%), while 126 participants had performed less than 50 procedures (47.7%).

**Table 1:** Subdivision according to surgical specialty.

Specialty	Number (*N*°)	Percentage (%)
General surgery	119	39.7
Obstetrics and gynecology	127	42.3
Urology	8	2.7
Otolaryngology-head and neck surgery (OHNS)	8	2.7
Plastic surgery	7	2.3
Vascular surgery	6	2.0
Thoracic surgery	20	6.7
Orthopedics	5	1.6
Total	300	100

**Table 2:** Subdivision according to regions where respondents are employed.

Region	Number (*N*°)	Percentage (%)
**Friuli Venezia Giulia**	74	24.8
Lombardia	58	19.4
Toscana	28	9.4
Lazio	28	9.4
Emilia Romagna	20	6.7
Sicilia	15	5.0
Piemonte	13	4.3
Liguria	9	3.0
Veneto	9	3.0
Abruzzo	9	3.0
Puglia	6	2.0
Calabria	5	1.7
Campania	4	1.3
Marche	3	1.0
Sardegna	3	1.0
Umbria	3	1.0
Basilicata	2	0.7
Trentino Alto Adige	1	0.3
Molise	0	0
Valle D’Aosta	0	0
Total	300	100

**Table 3:** Characteristics of our population.

	Number (*N*°)	Percentage (%)
**Gender**		
Female	147	49.2
Male	152	50.8
Missing data	1	
**Age (years)**		
20–30	100	33.4
31–40	138	46.2
41–50	44	14.7
51–60	16	5.4
Over 60	1	0.3
*Missing data*	1	
**Role of surgeon**		
Surgeon in specialized training	138	46.0
Surgeon	162	54.0
**Number of surgeries in the last 12 months**		
<50	110	41.8
50–100	74	28.1
>100	79	30.1
*Missing data*	37	
**Number of surgeries as first operator**		
<5	126	47.7
50–100	30	11.4
101–150	13	4.9
>150	95	36.0
Missing data	36	

### 3.1 Role of training in our surgical population

Analyzing the participants’ background, 61.7% of the respondents had laparoscopic training during their training and only 53.1% had a pelvic trainer during their residency (see Table 4). Regarding the opinion on the usefulness of laparoscopic training during their formation, 93.8% stated that it would be extremely useful. Concerning the activities used to implement their laparoscopic skills, 69.2% of the participants stated that they directly used the operating room while only 31.2% used the pelvic trainer, 29.2% webinars, 9.6% the virtual simulator and 4.1% no method. Among the most used surgical simulators we found the bench-top (53.6%), surgical pads (37.6%), cadaver-lab (28.5%), robotic simulator (24.4%) and virtual reality (10.4%).

**Table 4:** Laparoscopic training and its reported utility.

	Number (*N*°)	Percentage (%)
**Laparoscopic training**		
Yes	163	61.7
No	101	38.3
Missing data	36	
**Presence of a pelvic trainer during training**		
Yes	136	53.1
No	120	46.9
Missing data	44	
**Methods used to improve laparoscopic skills**		
Surgery in OR	180	69.2
Webinar	76	29.2
Pelvic trainer	83	31.9
Simulation courses	43	16.5
Virtual Simulator	25	9.6
Other	35	13.5
Nothing	11	4.1
Missing data	40	
**Reported usefulness of laparoscopic training during training**		
Yes	242	93.8
No	16	6.2
Missing data	42	
**Types of tested simulators** ^a^		
Surgical pads	83	37.6
Cadaver-lab	63	28.5
Bench-top/laparoscopic box simulator	119	53.6
Virtual reality	23	10.4
Robotic surgery simulator	54	24.4
Missing data	79	

^a^Multiple-choice answer.

### 3.2 Ergonomics and injuries in our surgical population

**Figure 1:**
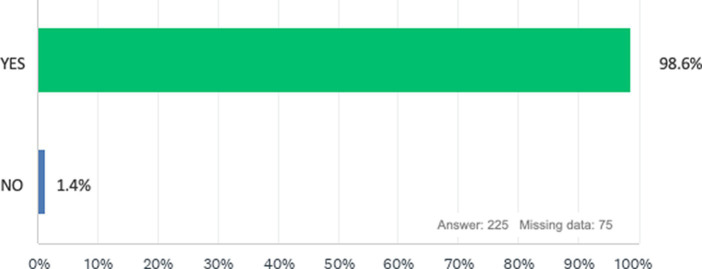
Have you ever had the feeling that your body was in an uncomfortable position in the operating room causing discomfort, muscle aches or pain during retraction, assistance or surgery?

**Figure 2:**
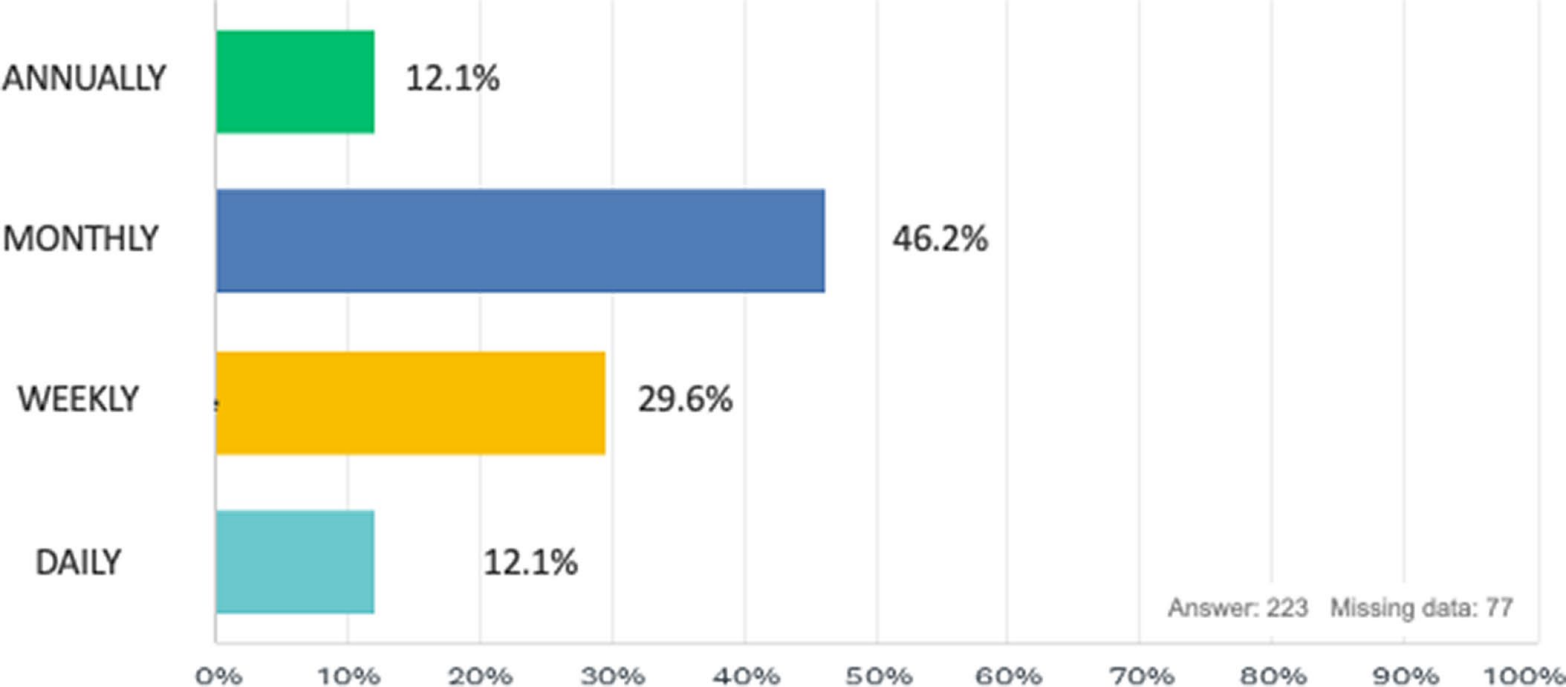
How often do these pains occcur?

**Figure 3:**
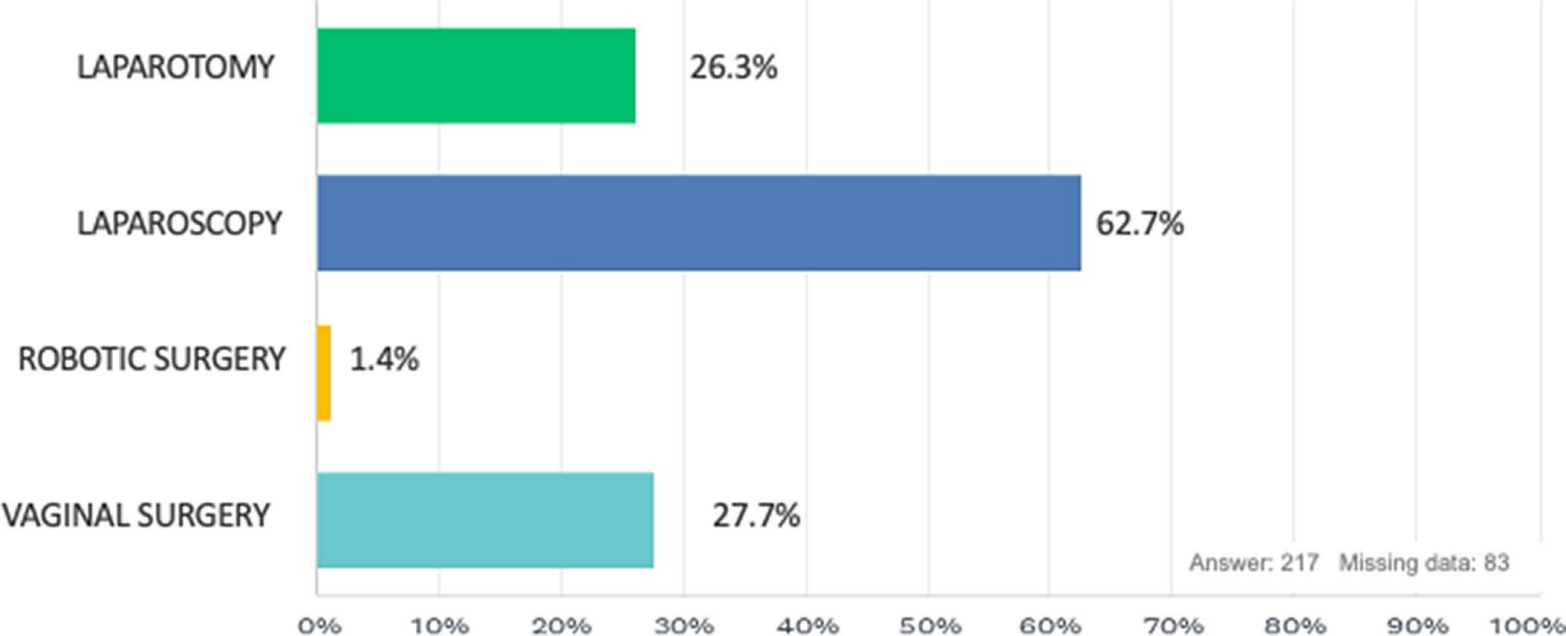
Which surgical approach causes you the most discomfort?

**Figure 4:**
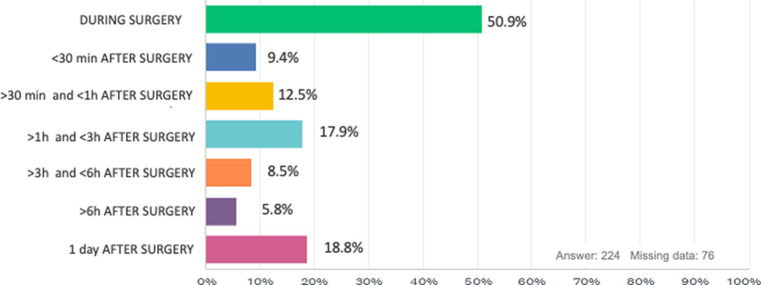
Estimation of the time of onset of symptoms.

In accordance with 98.7% of the respondents, during surgery we have the feeling of being in an uncomfortable position that causes discomfort or muscle pain (Figure 1), and regarding the frequency of these discomforts, the majority of our study population experiences these problems monthly (46.2%), while in 29.6% it is experienced weekly, 12.1% annually and finally 12.1% daily (Figure 2). The surgical approach that is most correlated with these disorders is laparoscopy (62.7%) while the one that causes the least discomfort is robotic surgery (1.4%; Figure 3). Regarding the onset from surgery, problems occur most immediately during the surgical procedure (50.9%) or within 3 h after surgery (Figure 4).

These discomforts cause 43.9% of our population to take a break or do short exercises to reduce pain during surgery, and the body areas most affected are the back (61.6%), neck (40.6%) and shoulders (37.8%). 60.4% also report a correlation of their discomfort with the arrangement of monitors in the operating room.

Regarding the use of devices to reduce these discomforts, 64.6% of the population reports that they do not use any while only 31.2% use elastic stockings and 2.7% use a corset. In addition, cases of true muscle injuries were also reported in 4.5% of the population that in 80% of cases led to discontinuation of clinical or surgical practice and in 6.8% of cases required abstention from work. In addition, in our population 9.4% stated that their knowledge of ergonomics in the operating room was sufficient to avoid problems or discomfort, and as many as 15.8% stated that they would change their surgical specialty in favor of specialists who are less physically straining (Tables 5A,B).

**Table 5:** Ergonomic data and pain. (A) pt.1. (B) pt.2.

A. Pt.1		
	Number (*N*°)	Percentage (%)
**Need to stop for a short rest or do exercises to relieve pain during surgery**		
Yes	98	43.9
No	125	56.1
Missing data	77	
**Body area of pain** ^a^		
Neck	91	40.6
Shoulders	85	37.8
Back	138	61.6
Hips	9	4.0
Legs	42	18.8
Feet	10	4.5
Arms	49	21.9
Hands	34	15.2
Missing data	76	
**Methods used to improve laparoscopic skills**		
Surgery in OR	180	69.2
Webinar	76	29.2
Pelvic trainer	83	31.9
Simulation courses	43	16.5
Virtual Simulator	25	9.6
Other	35	13.5
Nothing	11	4.1
Missing data	40	
**Reported correlation between monitor layout and neck pain**		
Yes	134	60.4
No	88	39.6
Missing data	78	
**Devices used to reduce pain** ^a^		
Elastic socks	69	31.2
Thigh-highs	0	0.0
Anklets	1	0.5
Kinesiology tape	1	0.5
Ergonomic insoles	1	0.5
Corset	6	2.7
Nothing	145	64.6
Missing data	79	

B. Pt.2		
	Number (*N*°)	Percentage (%)
**Reported cases of musculoskeletal injury in the operating room**		
Yes	10	4.5
No	213	95.5
Missing data	77			A. Pt.1			Number (*N*°)	Percentage (%)
**Cases in which intra-operative injury prevented clinical or surgical tasks from being performed**		
Yes	8	80.0
No	2	20.0
**Absence from work due to surgery injuries**		
Yes	15	6.8
No	206	93.2
Missing data	79	
**Reported use of strategies to reduce the risk of injury**		
Yes	68	30.5
No	155	69.5
Missing data	77	
**Surgeons' thoughts on ergonomics training in the operating room and whether it is sufficient**		
Yes	21	9.4
No	202	90.6
Missing data		
**Surgeons who would change specialities because of the reduced comfort of certain types of operations**		
Yes	35	15.8
No	187	84.2
Missing data	78	

^a^Multiple-choice answer.

## 4 Discussion

Our survey reveals that the perception of incorrect body position in the operating room and related physical disorders are extremely common among Italian surgeons, with 99% of surgeons reporting this perception in our study. These data highlight the need to improve ergonomic education in our surgeons during their training and to determine strategies that can avoid or reduce work-related injuries in the operating room. The purpose of our study was to investigate the availability of laparoscopic training and ergonomics training of Italian surgeons and to assess whether they suffer from physical complaints related to clinical practice during surgical activities. Our rates of musculoskeletal disorders are similar to those already reported in the scientific literature (5), and the most affected areas were the neck, shoulders, and low back. As reported in our survey, among the different surgical approaches the one most associated with discomfort is laparoscopy, reported by 63% of our participants, and this data is in line with those found in the literature. Due to previous studies, it is already known that the trapezius, deltoid and inferior spinal erector muscles are the ones most subjected to load from laparoscopy (25), which can lead to muscle injury or fatigue. The laparoscopic surgeon’s posture is characterized by increased elbow flexion with biceps activation and wrist flexion with ulnar carpal flexor activation (26). Non-ergonomic postures assumed in laparoscopic surgery can lead to an increased risk of fatigue during procedures and injury (27) while correct positioning, as proven in a 2012 study (28), may be associated with improved procedure performance, reduced excessive joint movements, and therefore lower risk of injury. Another interesting finding relates to laparoscopic training and ergonomics education in the operating room, which 94% of participants would have found useful during their specialized training and 61.7% of the respondents had laparoscopic training during their training. To date, there is little data available about ergonomic training programs in surgery and how they may affect surgical outcomes. A study performed by Franasaik et al. (29) developed a surgical ergonomics project for robotic surgery to demonstrate correct body positioning and strategies to avoid incorrect postures. The results of this study revealed that 88% of participants corrected their habits in the operating room and 74% reduced their physical stress during robotic surgery after taking part in the training course. Among surgery ergonomics programs, the one at Duke University is one of the few that focuses on residents in that it allows younger residents and students to be assisted and observed by older residents and also they are equipped with ergonomic lenses and have to use microbreaks and anti-fatigue mats during operations (30). In addition, another key role is assumed by ergonomics and the almost total absence of education during specialty training and consequent assumption of incorrect postures or attitudes during surgical practice. Lessons in surgical ergonomics can focus on explaining what are the main causes of physical stress and injuries (e.g., non-ergonomic postures, prolonged maintenance of the same posture, repeated physical stress) and showing what exercises can prevent these injuries (e.g., exercises to increase grip strength, wrist extension and/or flexion, or exercises to increase muscle strength). Our survey represents a picture of the current state of surgical education in Italy and of disorders related to the surgical profession, on which attention is rarely focused. There is now an urgent need for ergonomics in the operating room to be included within the training program of residents in order to better prepare surgeons for surgeries and prolong their careers, and for a reminder of proper postural habits to be included during surgical time-out to ensure proper operating room setup and improve surgical team members’ adherence to ergonomic principles.

Also, the importance of trainees’ opinions and benchmarks in laparoscopy is crucial for the continuous improvement of surgical education and practice. Several studies highlight this significance and underscore the value of incorporating feedback and structured training programs to enhance the skills and confidence of surgical trainees.

A study by De Franciscis et al. (31) emphasizes the need to assess the effects of tissue sealers in minor laparoscopic procedures. This prospective cohort study involving obstetrics and gynecology residents shows that trainee feedback is essential for evaluating new surgical technologies and their practical applications in clinical settings. By incorporating trainees’ perspectives, the study aims to enhance surgical outcomes and training efficiency.

Additionally, the development of a standardized laparoscopy curriculum for gynecology residents, as outlined by Shore et al. (32), demonstrates the critical role of trainee input in designing educational programs. This study used a Delphi approach to reach a consensus among experts, ensuring that the curriculum meets the educational needs of trainees and aligns with current surgical standards. The involvement of trainees in this process helps create a more relevant and effective training program.

Further validation of this standardized curriculum was conducted through a randomized controlled trial by Shore et al. (33), which highlighted the importance of structured training and assessment. This study confirmed that a well-designed curriculum improves laparoscopic skills and confidence among gynecology residents. The inclusion of trainee feedback in the validation process ensures that the curriculum addresses the practical challenges faced by residents during their training.

Regarding the powers of our paper, one of the primary strengths is its comprehensive data collection. By including 300 participants, the survey ensures a robust sample size that enhances the reliability and generalizability of its findings. The detailed demographic breakdown, with Gynecology and Obstetrics (42.3%) and General Surgery (39.7%) being the most represented specialties, offers targeted insights into the specific ergonomic challenges these surgeons face. Additionally, the survey highlights significant gaps in training, revealing that 61.7% had laparoscopic training and only 53.1% had access to a pelvic trainer during residency. This information is crucial for improving surgical education and training programs.

Another notable strength is the detailed reporting of ergonomic issues. The survey indicates that 98.7% of respondents experience discomfort or muscle pain during surgery, with varying frequencies of discomfort (monthly, weekly, annually, daily). This nuanced data underscores the prevalence and severity of ergonomic problems in the surgical field.

Despite its strengths, the survey has some notable weaknesses. The reliance on self-reported data introduces potential bias, as participants might underreport or overreport their experiences and discomfort levels. The absence of objective ergonomic assessments limits the precision and quantifiability of the data on physical strain experienced by surgeons.

Additionally, the variability in training experiences among respondents indicates a lack of standardized training programs, which could affect the consistency of the data. The focus on Gynecology, Obstetrics, and General Surgery, while providing specific insights, excludes other specialties that may also face significant ergonomic challenges.

Despite this, our survey allows us to highlight some now-known gaps present in the surgical training program of our schools and the lack of protection toward our surgeons during their long career. Certainly in support of this thesis, further studies will be needed to ascertain internationally the shortcomings in surgical training and injury rates related to operating room practice, and to this end, a new survey with worldwide dissemination is currently being conducted (ERGO Study) (34), whose data are currently being analyzed. The care of patients is essential but, to be able to treat them in the best manner possible, our health and physical care should also be a priority, and to achieve this target, technology should also be a valuable ally, always trying to use it in the best possible way.

## Data availability statement

The raw data supporting the conclusions of this article will be made available by the authors, without undue reservation.

## Author contributions

SR: Writing – review & editing. MD'I: Writing – original draft, Data curation. FP: Data curation, Investigation, Validation, Writing – review & editing. MA: Conceptualization, Formal analysis, Visualization, Writing – review & editing. VChe: Conceptualization, Visualization, Writing – review & editing. MP: Validation, Visualization, Writing – original draft. ACa: Supervision, Visualization, Writing – original draft. SC: Supervision, Visualization, Writing – original draft. GP: Investigation, Methodology, Writing – original draft. RM: Investigation, Supervision, Writing – original draft. ACo: Validation, Visualization, Writing – original draft. VChi: Supervision, Visualization, Writing – original draft. GS: Supervision, Visualization, Writing – original draft. LD: Supervision, Visualization, Writing – original draft. GV: Data curation, Formal analysis, Supervision, Visualization, Writing – original draft.

## SPIGC working group

Collaborators to be indexed:

*Executive Committee*: Federico Berton, Luigi Conti, Giampaolo Formisano, Eleonora Guaitoli, Angelo Iossa, Michele Maruccia, Andrea Mazzari, Luigi Oragano, Alessandro Pasculli, Francesca Ratti, Matteo Serenari, Alberto Settembrini, Pasqualino Sirignano, Domenico Soriero, Carlo Vallicelli.

*Regional Lead*: Stefano Cianci (Sicilia), Giulia De Iaco (Puglia), Francesca Falcone (Campania), Sara Giaccari (Triveneto), Marco Giovenzana (Lombardia), Edoardo Pasqui (Toscana), Marco Petrillo (Sardegna), Luca Portigliotti (Piemonte), Giuseppe Sena (Calabria), Marco Sparavigna (Liguria).

*Dissemination Committee*: Giordana Bettini, Gianfranco Fanello, Paolo Mendogni, Lorenzo Monteleone, Davide Pertile Nicoletta Pia Ardò, Pasquina Tomaiuolo, Sara Negrello, Mattia Di Bartolomeo, Romeo Patini, Alberto Vito Marcuzzo, Alberto Campione, Giovanni Comacchio, Giacomo Murana, Martino Antonio, Mattia Manitto, Giuseppe Galzerano, Carlo Di Marco, Francesco Velluti, Gianmauro Berardi, Andrea Romboli, Jacopo Weindelmejer, Domenico Tamburrino, Alessandro Calarco, Luigi Losco, Eleonora Nacchiero, Rossella Elia, Federico Lo Torto, Giovanni Vicenti, Vincenzo Pappalardo, Dafne Pisani, Graziano Palmisano, Debora Brascia, Luigi Troisi, Federica Renzi, Fabio Melandro, Silvia Pecere, Carlo Gazia, Gregorio Di Franco, Gaetano Romano, Alberto Bolletta, Emanuele Botteri, Giovanna Di Meo, Carlo Ronsini, Sonia Chiappetta, Ilaria Sgaramella, Francesco Pennestri, Antonella Girardi, Donatella Mariniello, Marco Marcasciano, Michele Telegrafo, Simona Fragomeni, Francesca De Paoli, Giorgio Bogani, Salvatore Gueli Alletti, Luigi Pedone Anchora, Luigi Della Corte, Elisa Piovano, Martina Borghese, Cristina Taliento, Diego Raimondo, Antonio Raffone, Jvan Casarin, Emanuele Perrone, Guglielmo Stabile, Vito Capozzi.
