# Supplementary material for: Ergonomics in the operating room and surgical training: a survey on the Italian scenario
Source: Front Public Health. 2024 Aug 7;12:1417250. doi: 10.3389/fpubh.2024.1417250 (PMC11337608; doi:10.3389/fpubh.2024.1417250)
Supplement: Supplementary file 1 [file Data_Sheet_1.PDF]

Ergonomia in sala operatoria

Informazioni generali

1. Genere

◯ Donna

◯ Uomo

2. Fascia di età

◯ 20-30

◯ 31-40

◯ 41-50

◯ 51-60

◯ Oltre 60

3. Ruolo

◯ Medico in formazione specialistica

◯ Medico strutturato

◯ Altro (specificare)

[ ]

4. Città della sede lavorativa

[ ]

5. Specializzazione

◯ Chirurgia generale

- ◯ Ginecologia e Ostetricia
- ◯ Urologia
- ◯ Otorinolaringoiatria
- ◯ Neurochirurgia
- ◯ Chirurgia plastica
- ◯ Chirurgia vascolare
- ◯ Altro (specificare)

Succ.

Gestito da
SurveyMonkey
Scopri come è facile creare un'indagine.

Informativa sulla privacy e Informativa sui cookie

Ergonomia in sala operatoria

Esperienza e training laparoscopico

6. Quanti interventi hai eseguito negli ultimi 12 mesi?

- ◯ Meno di 50
- ◯ Fra 50 e 100
- ◯ Oltre 100

7. Quanti interventi da primo operatore hai eseguito nel corso della tua carriera?

- ◯ <50
- ◯ 50-100
- ◯ 101-150
- ◯ Oltre 150

8. Hai mai eseguito un training in laparoscopica?

- ◯ si
- ◯ no

9. Quali metodi utilizzi per incrementare le tue abilità laparoscopiche?

- ☐ Interventi in sala operatoria
- ☐ Webinar

- [ ] Pelvic trainer
- [ ] Corsi di simulazione
- [ ] Simulatore virtuale
- [ ] Nessuno
- [ ] Altro (specificare)

10. Quanto spesso riesci ad esercitarti nelle procedure laparoscopiche in un mese?

- ◯ <5
- ◯ 5-10
- ◯ 11-15
- ◯ 16-20
- ◯ 21-25

11. Durante la formazione specialistica hai avuto accesso al pelvic trainer?

- ◯ si
- ◯ no

12. Ritieni sarebbe stato utile eseguire un training laparoscopica nella tua formazione specialistica?

- ◯ si
- ◯ no

13. Quale tipo di simulatore hai mai provato?

- [ ] Surgical pads
- [ ] Cadaver-lab
- [ ] Bench-top o laparoscopic box simulator
- [ ] Realtà virtuale
- [ ] Simulatore di chirurgia robotica

Prec. Succ.

Gestito da
SurveyMonkey
Scopri come è facile creare un'indagine.

Informativa sulla privacy e Informativa sui cookie

Ergonomia in sala operatoria

Ergonomia

14. Ha mai avuto la sensazione che il tuo corpo si trovasse in una posizione scomoda in sala operatoria causando disagio, dolori muscolari o dolori durante la ritrazione, l'assistenza o l'intervento?

- ◯ si
- ◯ no

15. Con quale frequenza si verificano questi dolori?

- ◯ Annualmente
- ◯ Mensilmente
- ◯ Settimanalmente
- ◯ Quotidianamente

16. Quale approccio chirurgico ti reca maggiori disturbi?

- ☐ Laparotomico
- ☐ Laparoscopico
- ☐ Robotico
- ☐ Vaginale

17. Si prega di stimare il tempo di insorgenza dei sintomi?

- ☐ Durante l'intervento
- ☐ < 30 min dopo l'intervento
- ☐ da 30min a 1 h dopo l'intervento
- ☐ 1-3h dopo l'intervento
- ☐ 3-6h dopo l'intervento
- ☐ 6h dopo l'intervento
- ☐ il giorno successivo

18. Durante un intervento hai mai avuto la necessità di fermarti per un breve riposo o fare degli esercizi per alleviare i dolori?

- ◯ si
- ◯ no

19. Quali sono le aree del corpo che ti danno più fastidio?

- ☐ Collo
- ☐ Spalle
- ☐ Schiena
- ☐ Fianchi
- ☐ Gambe
- ☐ Piedi
- ☐ Braccia
- ☐ Mani

20. Quanti monitor ci sono nella tua sala operatoria?

- ◯ 1
- ◯ 2
- ◯ 3
- ◯ 4

21. Come sono disposti i monitor nella tua sala operatoria?

22. Noti una correlazione fra la disposizione dei monitor o alcune procedure chirurgiche con la comparsa di dolore cervicale?

- ◯ si
- ◯ no

23. Usi qualche dispositivo specifico per prevenire l'insorgenza di questi disturbi?

- ☐ Calze elastiche a compressione
- ☐ Cosciali
- ☐ Cavigliere
- ☐ Nastro kinesologico
- ☐ Nessuno
- ☐ Altro (specificare)

24. L'uso di attrezzature chirurgiche specifiche provoca sintomi con maggiore frequenza?

- [ ] Caschetto chirurgico con faro
- [ ] Robot
- [ ] Attrezzature laparoscopiche
- [ ] Lenti
- [ ] Microscopio
- [ ] Altro (specificare)

25. Ha mai subito una lesione muscolo-scheletrica in sala operatoria?

- ◯ si
- ◯ no

26. Questa lesione le ha impedito di svolgere le mansioni cliniche o chirurgiche?

- ◯ si
- ◯ no

27. L'infortunio ha impedito di svolgere attività al di fuori dell'ospedale o della clinica?

- ◯ si

◯ no

28. Si è mai assentato dal lavoro a causa di dolori/lesioni muscoloscheletriche?

◯ si

◯ no

29. Si è mai recato da uno dei seguenti medici per un trattamento o un consulto?

☐ Massaggiatore

☐ Personal trainer

☐ Fisioterapista

☐ Ortopedico

☐ Fisiatra

☐ Osteopata

☐ No

☐ Altro (specificare)

30. Attuate abitualmente strategie di riduzione del rischio in sala operatoria per alleviare i problemi muscoloscheletrici?
Per esempio: sgabelli, tappetini per stare in piedi, pause temporizzate, stretching intraoperatorio.

◯ si

◯ no

31. Esegue abitualmente stretching o esercizi per gruppi muscolari specifici al di fuori della sala operatoria per migliorare le aree problematiche?

- ◯ si
- ◯ no

32. Ha seguito una formazione formale, lezioni o discussioni sulle seguenti aree?

- ☐ Postura/meccanica del corpo in sala operatoria
- ☐ Posizione corretta della testa, spalle, colonna vertebrale e bacino
- ☐ Impostazione ergonomica al tavolo operatorio o alla consolle robotica
- ☐ Nessuna delle precedenti

33. Ritiene che la formazione degli studenti di medicina e degli specializzandi di chirurgia in queste aree sia appropriata/sufficiente?

- ◯ si
- ◯ no

34. Quali suggerimenti avete per migliorare la longevità e il benessere fisico dei futuri chirurghi? Ad esempio: una migliore

formazione in materia di ergonomia e postura, l'osservazione diretta osservazione diretta/video ripresa e valutazione delle abitudini in sala operatoria, accesso alla fisioterapia in ospedale, massoterapia fisioterapia in ospedale, massoterapia, non far spostare/sollevare i pazienti da chirurghi/residenti (personale dedicato ai traslochi).

35. Il ridotto comfort di alcuni tipi di interventi ti ha mai fatto pensare di cambiare specialità o ambito di interesse a favore di una chirurgia meno impegnativa?

- ◯ si
- ◯ no

36. Hai mai sofferto nella tua carriera o soffri di algie mosucoloscheletriche ?

- ◯ si
- ◯ no

37. Hai perso giornate lavatorative a causa di problematiche osteartromuscolari legate alla posizione in sala operatoria?

◯ si

◯ no

Prec. Fine

Gestito da
SurveyMonkey
Scopri come è facile creare un'indagine.
